# Fukutin Protein Participates in Cell Proliferation by Enhancing Cyclin D1 Expression through Binding to the Transcription Factor Activator Protein-1: An In Vitro Study

**DOI:** 10.3390/ijms222212153

**Published:** 2021-11-10

**Authors:** Yukinori Okamura, Tomoko Yamamoto, Ryota Tsukui, Yoichiro Kato, Noriyuki Shibata

**Affiliations:** Division of Human Pathology & Pathological Neuroscience, Department of Pathology, Graduate School of Medicine, Tokyo Women’s Medical University, Tokyo 162-8666, Japan; yamamoto.tomoko@twmu.ac.jp (T.Y.); tsukuryo0525@gmail.com (R.T.); katoyo@twmu.ac.jp (Y.K.); shibatan@twmu.ac.jp (N.S.)

**Keywords:** activator protein-1, astrocytes, cell proliferation, c-Jun, cyclin D1, fukutin, Fukuyama congenital muscular dystrophy

## Abstract

The causative gene of Fukuyama congenital muscular dystrophy (fukutin) is involved in formation of the basement membrane through glycosylation of alpha-dystroglycan. However, there are other proposed functions that have not been fully understood. Using cultured astrocytes (1321N1), we found nuclear localization of fukutin and a positive relationship between fukutin expression and cell proliferation. Among potential proteins regulating cell proliferation, we focused on cyclin D1, by reverse-transcription polymerase chain reaction, Western blotting, immunocytochemistry, enzyme-linked immunosorbent assay (ELISA), and sandwich ELISA. Expression of cyclin D1 was significantly downregulated by fukutin knockdown and significantly upregulated by fukutin overexpression. Moreover, fukutin was proven to bind to the activator protein-1 (AP-1) binding site of cyclin D1 promoter, as well as the AP-1 component c-Jun. The c-Jun phosphorylation status was not significantly influenced by knockdown or overexpression of fukutin. The present results provide in vitro evidence for a novel function of fukutin, which participates in cell proliferation by enhancing cyclin D1 expression through forming a complex with AP-1. It is likely that fukutin is a potential cofactor of AP-1.

## 1. Introduction

Fukuyama-type congenital muscular dystrophy (FCMD) is an inherited disease that is characterized by congenital anomalies of the central nervous system (CNS) and eye, as well as myopathy [1,2]. Previously, fukutin was identified as a causative gene of FCMD [3]. Initial studies focused on implications for fukutin protein in the glycosylation of alpha-dystroglycan (α-DG), which bears close relevance to formation of the basement membrane [4,5]. However, fukutin functions except for α-DG glycosylation have not been fully characterized by molecular biological approaches. In the CNS of patients with FCMD, hypoglycosylation of α-DG due to downregulation of fukutin is responsible for impaired basement membrane formation of the glial limitans, leading to congenital malformations, including polymicrogyria [6]. Since the glia limitans originates from foot processes of astrocytes, it is evident that altered astrocytes give rise to the formation of CNS lesions in FCMD [7,8].

On the other hand, fukutin is expressed in various organs and tissues in the body [9,10], as well as in cells other than astrocytes in the CNS [8,9,11]. It has been reported that the expression level of fukutin varies among different histological types and grades of gastric cancer [12]; in particular, fukutin expression in gastric carcinomas arising in atomic bomb survivors was more upregulated in the high-radioactivity-exposure group [13]. These observations suggest a role for fukutin in cell proliferation ability. In relation to this, it is of interest that fukutin is localized in the nucleus of HeLa cells and breast cancer cell lines [8,10].

It is accepted worldwide that cyclin D1 is an essential subunit protein of the cell-cycle engine during transition from phase G1 to phase S as the rate-limiting step of the cell cycle [14,15]. Transcription of cyclinD1 is triggered by the promoter binding to several transcription factors, including activator protein-1 (AP-1), nuclear factor-kappa B (NF-κB), E2F, and Oct-1 [16]. AP-1 is a heterodimeric protein composed of c-Fos and c-Jun [17,18,19,20]. It is known that AP-1 upregulates the expression of genes that govern cell proliferation, intercellular communication, amplification and diffusion of pathogenic signals, inflammatory reaction, and initiation and promotion of tumorigenesis [21,22]. Moreover, Oak et al. demonstrated that the α-DG signaling phosphorylates the downstream enzyme c-Jun N-terminus kinase (JNK) [23] that catalyzes the conversion of c-Jun to phosphorylated c-Jun (p-c-Jun), leading to activation of AP-1 [17,18,19,20]. Given these backgrounds, it is hypothesized that fukutin may regulate cell proliferation through the AP-1-mediated cyclin D1 induction system. To test this hypothesis, we investigated the molecular biological mechanisms via which fukutin exerts cell proliferation ability using molecular biological approaches, including reverse-transcription polymerase chain reaction (RT-PCR) analysis and enzyme-linked immunosorbent assay (ELISA), focusing on astrocytes, the most common cell type of the CNS.

## 2. Results

### 2.1. Fukutin Is Localized in Both the Nucleus and Cytoplasm

**Figure 1:**
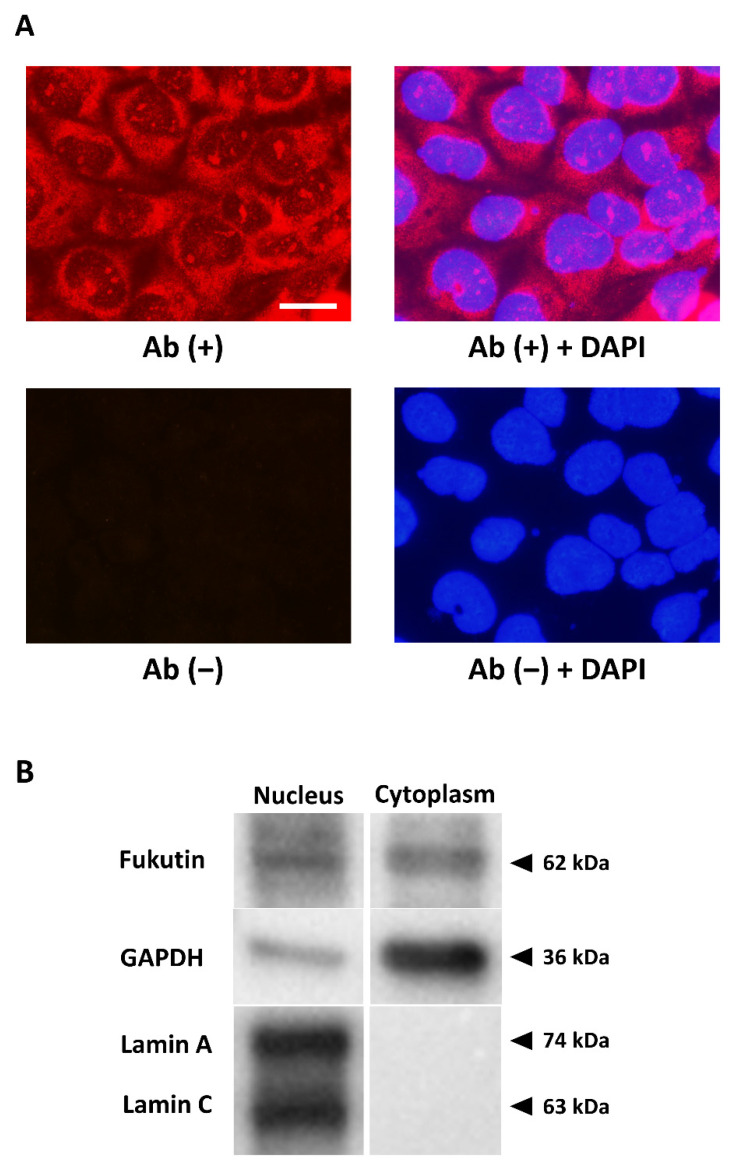
The expression status of fukutin protein in 1321N1 cells using an anti-fukutin antibody. (**A**) Immunofluorescence staining with the antibody in cells cultured on slides reveals that the immunoreactivity is localized not only in the cytoplasm with Cy3 (red) but also in the nucleus, with the latter proven by merging (purple) with DAPI (blue). No immunoreactivity is visible on slides with omission of the primary antibody. Bars = 20 μm. (**B**) Western blotting with the antibody identifies immunoreactive signals in the nuclear and cytoplasmic fractions at 62 kDa by the chemiluminescence method. Lamin A/C and GAPDH are nuclear and cytoplasmic markers, respectively.

Fluorescence immunocytochemistry with the anti-fukutin antibody in 1321N1 cells cultured on chamber slides revealed that the immunoreactivity was localized in the nucleus, as well as the cytoplasm (Figure 1A). Slides from which the primary antibody was omitted did not show any immunoreaction product deposits, guaranteeing a negative reaction control. Western blotting with the anti-fukutin antibody identified fukutin immunoreactivity signals in the nuclear and cytoplasmic fractions at a predicted mobility (Figure 1B). Successful extraction of both fractions was verified by detecting immunoreactive determinants of the respective markers lamin A/C and GAPDH. Omission of the primary antibodies on blots served a negative reaction control (data not shown).

### 2.2. Knockdown and Overexpression of Fukutin Alter Proliferation Ability

**Figure 2:**
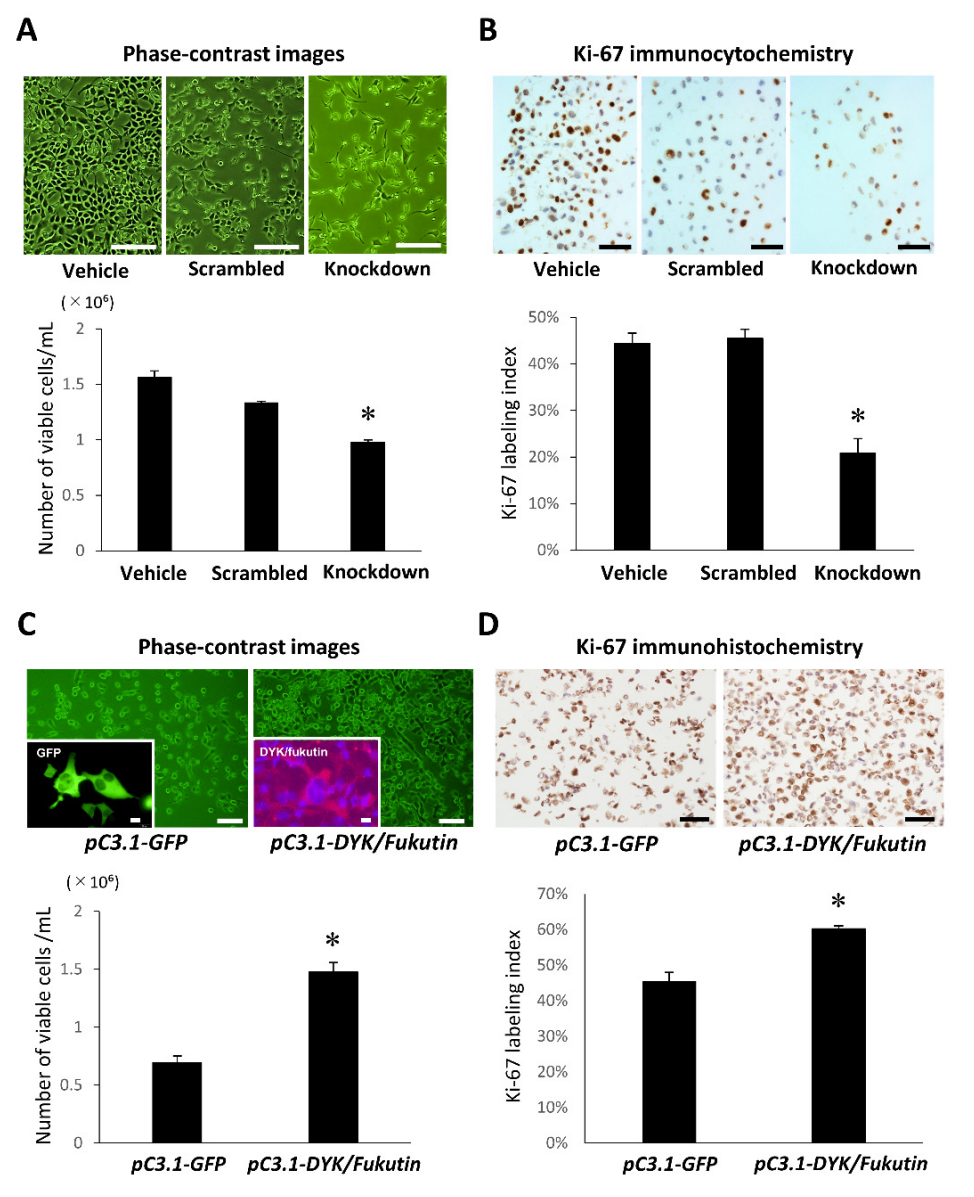
Influence of knockdown or overexpression of fukutin on proliferation ability of 1312N1 cells. (**A**) The density of viable cells determined using the cell counting kit is significantly reduced by fukutin knockdown (* *p* < 0.005 versus the vehicle and scrambled groups). Bars = 200 μm. (**B**) The Ki-67 labeling index is significantly reduced by fukutin knockdown. * *p* < 0.005 versus the vehicle and scrambled groups. Bars = 50 μm. (**C**) The density of viable cells determined using the cell counting kit is significantly increased by fukutin overexpression (* *p* < 0.005 versus the vehicle group). Insets indicate the presence of fluorescence signals for GFP (green) and immunofluorescence signals for DYK/fukutin (red); these observations prove a successful transfection with both genes. Bars = 100 μm and 20 μm (inset). (**D**) The Ki-67 labeling index is significantly increased by fukutin overexpression (* *p* < 0.005 versus the vehicle group). pC3-GFP, vehicle transgene construct; pC3-DYK/fukutin, transgene construct for fukutin overexpression. Bars = 50 μm. All experiments were conducted in triplicate.

Both the cell density (Figure 2A) and the Ki-67 labeling index (Figure 2B) were significantly reduced in the knockdown group as compared to the vehicle and scrambled groups. By contrast, the cell density (Figure 2C) and Ki-67 labeling index (Figure 2D) were significantly increased in the overexpression group as compared to the vehicle group. Successful transfection was proven by detecting fluorescence signals for GFP and immunofluorescence signals for DYK/fukutin.

### 2.3. Knockdown and Overexpression of Fukutin Alter Cyclin D1 Transcript and Protein Levels

**Figure 3:**
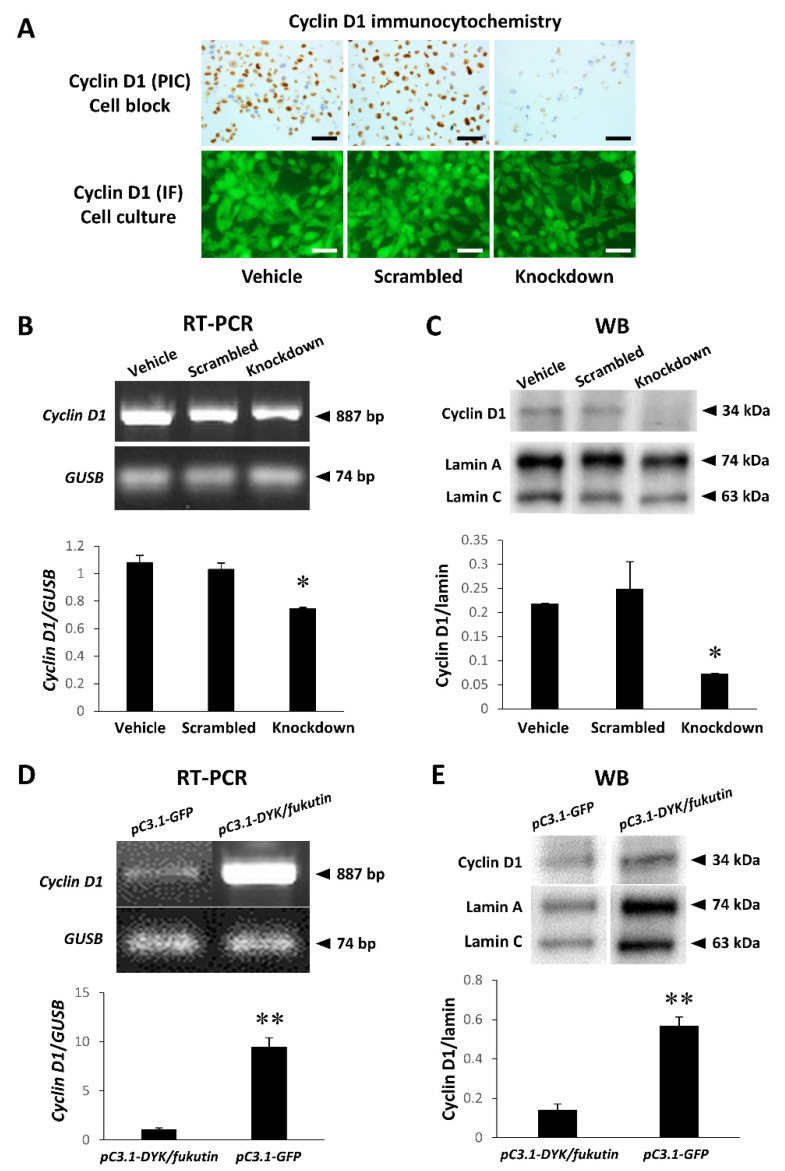
Influence of knockdown or overexpression of fukutin on the expression status of cyclin D1 transcript and protein in 1321N1 cells. (**A**) Immunocytochemical staining by the polymer immunocomplex and immunofluorescence methods with an anti-cyclin D1 antibody depicts a pronounced reduction in the number of immunoreactive cells in the fukutin knockdown group as compared to the vehicle and scrambled groups. Bars = 50 μm. (**B**) Semiquantitative RT-PCR analysis reveals a significant reduction in cyclin D1 transcript signals by fukutin knockdown (* *p* < 0.05 versus the vehicle and scrambled groups). (**C**) Western blot analysis identifies a significant reduction in cyclin D1-immunoreactive signals by fukutin knockdown (* *p* < 0.05 versus the vehicle and scrambled groups). (**D**) Semiquantitative RT-PCR analysis reveales a significant increase in cyclin D1 transcript signals by fukutin knockdown (** *p* < 0.005 versus the vehicle group). (**E**) Western blot analysis identifies a significant increase in cyclin D1-immunoreactive signals by fukutin knockdown (** *p* < 0.005 versus the vehicle group). pC3-GFP, vehicle transgene construct; pC3-DYK/fukutin, transgene construct for fukutin overexpression. All experiments were conducted in triplicate.

Immunocytochemistry with the anti-cyclin D1 antibody in 1321N1 cells cultured on chamber slides allowed visualizing nuclear localization of the immunoreactivity, and the cell density evidently appeared lower in the fukutin knockdown group as compared to the vehicle and scrambled groups (Figure 3A). Cyclin D1 transcript (Figure 3B) and protein (Figure 3C, Supplementary Materials Figure S1) levels were significantly reduced in the fukutin knockdown group as compared to the vehicle and scrambled groups. By contrast, cyclin D1 transcript (Figure 3D) and protein (Figure 3E, Supplementary Materials Figure S2) levels were significantly increased in the fukutin overexpression group as compared to the vehicle group.

### 2.4. Fukutin Protein Binds to the Promoter Region of Cyclin D1

**Figure 4:**
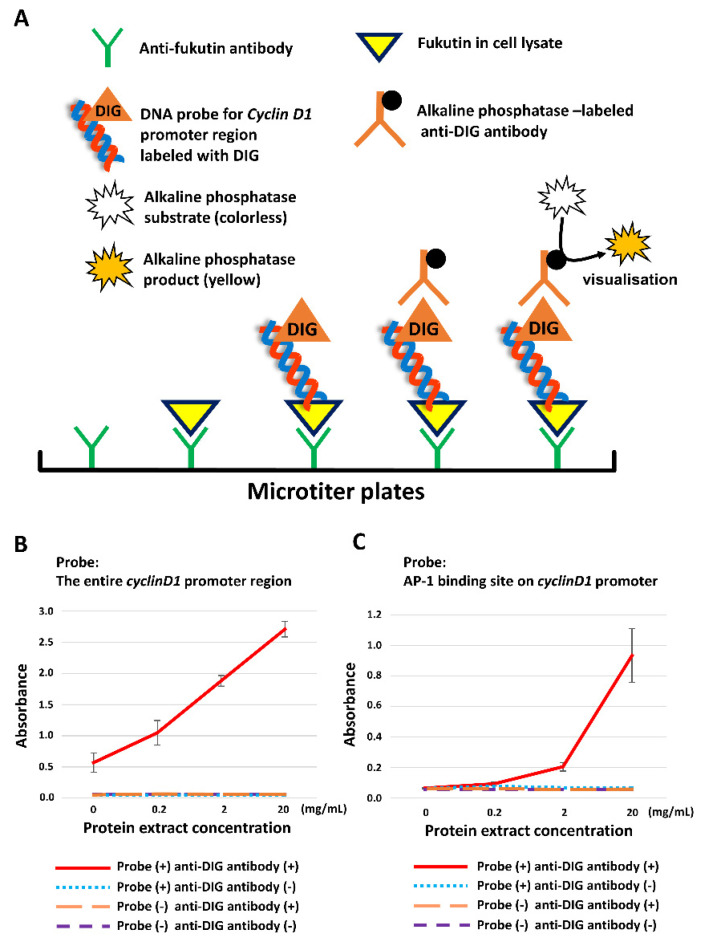
The principle (**A**) and results (**B**,**C**) of ELISA to validate fukutin binding to cyclin D1 promoter in total protein extracts obtained from 1321N1 cell lysate samples. (**A**) Prior to experiments, a microplate is coated with an anti-fukutin antibody. The antibody traps fukutin protein in the samples. Digoxigenin (DIG)-labeled oligonucleotide DNA probes, designated for the entire cyclin D1 promoter region and for the AP-1 binding site on the cyclin D1 promoter, are predicted to bind to the fukutin. Successful binding is detected with an alkaline phosphatase-labeled anti-DIG antibody and visualized using an enzyme reaction yellow product. The obtained values are subjected to statistical comparison. (**B**) The fukutin/entire promoter region complex levels are significantly increased in a manner dependent on cellular protein concentrations (*p* < 0.0001 on one-way ANOVA). (**C**) The fukutin/AP-1 binding site complex levels are significantly increased in a manner dependent on cellular protein concentrations (*p* < 0.0001 on one-way ANOVA). All experiments were conducted in triplicate.

The principle of ELISA is depicted in Figure 4A. First of all, the fukutin/entire cyclin D1 promoter region complex levels were significantly increased in a cellular protein concentration-dependent manner (Figure 4B). Next, the fukutin/5′-sided one-third portion of the promoter complex levels also showed a similar result (data not shown). On the basis these observations, we focused on the AP-1 binding site that is known to be located at the 5′-sided one-third portion of the promoter region [16]. As a consequence, the fukutin/AP-1 binding site complex levels were significantly increased in a manner dependent on cellular protein concentrations (Figure 4C).

### 2.5. Fukutin Protein Binds to the AP-1 Component c-Jun/p-c-Jun

**Figure 5:**
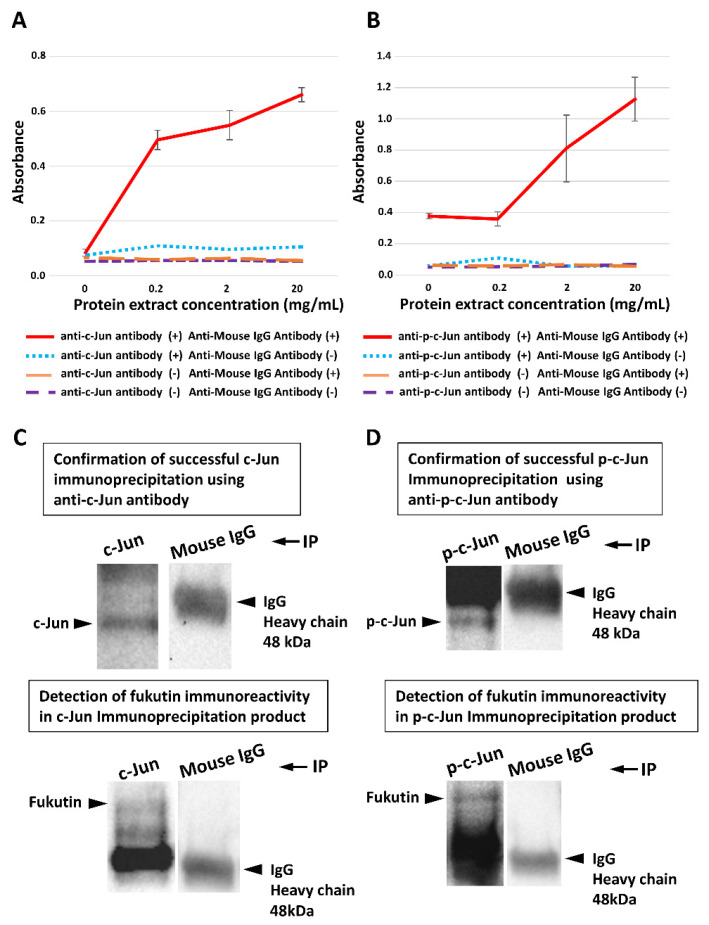
Results of sandwich ELISA (**A**,**B**) and Western blotting (**C**,**D**) to validate fukutin binding to the AP-1 component c-Jun/p-c-Jun in total protein extracts obtained from 1321N1 cell lysate samples. (**A**) The fukutin/c-Jun complex levels are significantly increased in a manner dependent on cellular protein concentrations (*p* < 0.0001 on one-way ANOVA). (**B**) The fukutin/p-c-Jun complex levels are significantly increased in a manner dependent on cellular protein concentrations (*p* < 0.0002 on one-way ANOVA). (**C**) A fukutin-immunoreactive signal is detected in immunoprecipitation products using an anti-c-Jun antibody but not in those using a nonspecific IgG. (**D**) A fukutin-immunoreactive signal is detected in immunoprecipitation products using an anti-p-c-Jun antibody but not in those using the nonspecific IgG. IgG, immunoglobulin G; IP, immunoprecipitation. All experiments were conducted in triplicate.

Sandwich ELISA disclosed that levels of the fukutin/c-Jun complex (Figure 5A) and the fukutin/p-c-Jun complex (Figure 5B) were significantly increased in a manner dependent on cellular protein concentrations. On Western blots, a fukutin-immunoreactive signal was detected in immunoprecipitants obtained with the anti-c-Jun antibody (Figure 5C) and with the anti-p-c-Jun antibody (Figure 5D), whereas no fukutin-immunoreactive signal was detected in immunoprecipitants obtained with a nonspecific IgG.

### 2.6. JNK Inhibition Downregulates Cyclin D1 Expression

**Figure 6:**
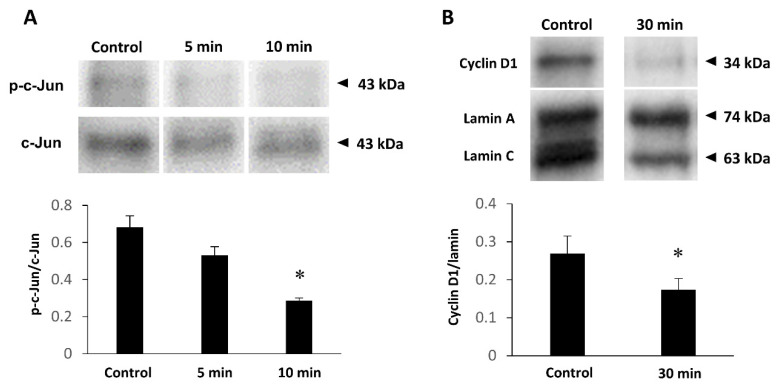
Western blots of 1321N1 cell lysate samples when using JNK inhibitor. (**A**) Relative values of p-c-Jun immunoreactivity normalized by c-Jun immunoreactivity are significantly reduced by adding the JNK inhibitor SP600125 (* *p* < 0.0002 versus the control group in ANOVA). (**B**) Relative values of cyclin D1 immunoreactivity normalized by lamin immunoreactivity are significantly reduced by adding the inhibitor (* *p* < 0.01 versus the control group in Student’s *t*-test). All experiments were conducted in triplicate.

Successful JNK inhibition was validated by Western blotting. Addition of JNK inhibitor SP600125 resulted in a significant reduction in the p-c-Jun/c-Jun optical density ratio (Figure 6A). Accordingly, we investigated the influence of JNK inhibition on cyclin D1 expression. As a consequence, the cyclin D1/lamin optical density ratio on blots was significantly reduced by JNK inhibition (Figure 6B).

### 2.7. Knockdown or Overexpression of Fukutin Does Not Affect Phosphorylation of JNK or c-Jun

**Figure 7:**
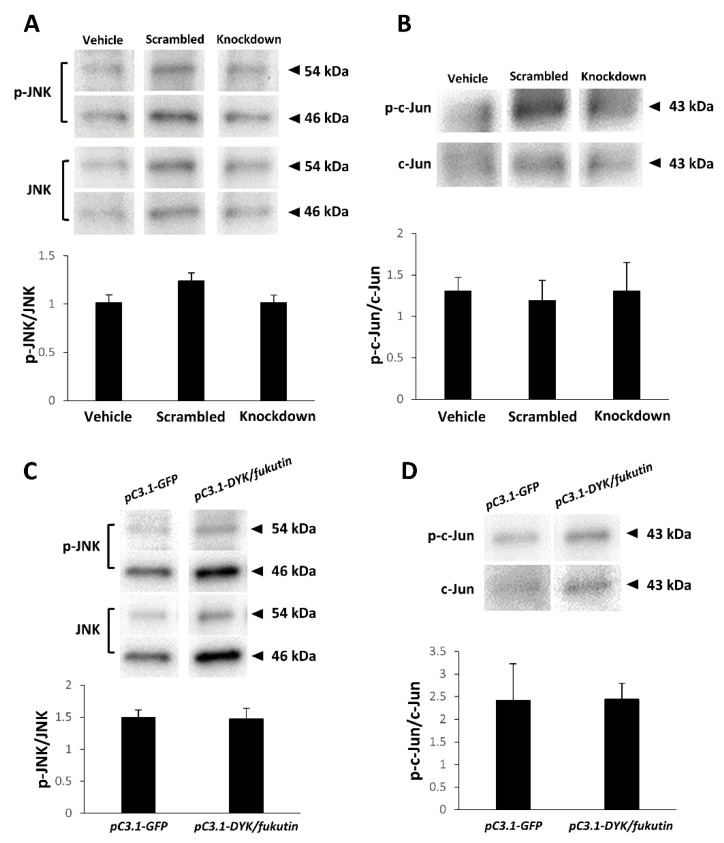
Influence of knockdown or overexpression of fukutin on phosphorylation of JNK and c-Jun on Western blots of 1321N1 cell lysate samples. (**A**,**B**) There is no significant difference in the p-JNK/JNK optical density ratio (**A**) and the p-c-Jun/c-Jun optical density ratio (**B**) among the vehicle, scrambled, and knockdown groups. (**C**,**D**) There is no significant difference in the p-JNK/JNK optical density ratio (**C**) and the p-c-Jun/c-Jun optical density ratio (**D**) between the vehicle and overexpression groups. pC3-GFP, vehicle transgene construct; pC3-DYK/fukutin, transgene construct for fukutin overexpression. All experiments were conducted in triplicate.

We investigated the influence of the fukutin expression status on the JNK and c-Jun phosphorylation status by Western blotting. Consequently, there was no significant difference in the p-JNK/JNK optical density ratio (Figure 7A) or the p-c-Jun/c-Jun optical density ratio (Figure 7B) among the vehicle, scrambled, and knockdown groups. Moreover, there was no significant difference in the p-JNK/JNK optical density ratio (Figure 7C) or the p-c-Jun/c-Jun optical density ratio (Figure 7D) between the vehicle and overexpression groups.

## 3. Discussion

Earlier studies documented that fukutin protein is localized in the Golgi apparatus [3,24], and some later investigators reported other intracellular localization patterns of fukutin in different cell types. Using immunocyto/immunohistochemical and immunoblotting approaches, fukutin has been shown to localize in the nucleus and cytoplasm, including the endoplasmic reticulum of HeLa cells [10] and of retinal cells [25]. Another study on myoblasts indicated that fukutin localizes in the Golgi apparatus in cells transfected with the wildtype fukutin and in the endoplasmic reticulum in cells transfected with mutant fukutin [24]. In the present study, we convincingly demonstrated the presence of fukutin protein in the nucleus, as well as the cytoplasm, of astrocytoma-derived 1321N1 cells, evidenced by immunocytochemistry on chamber slides and Western blotting of nuclear and cytoplasmic fractions. Since alternative splicing of fukutin has been reported [26], isoforms might be detected by immunocytochemistry. However, the authors consider the nuclear and cytoplasmic localization appropriate, since Western blotting showed a band indicating full-length fukutin in both the nucleus and the cytoplasm. On the other hand, it is well known that cyclin D1 is often overexpressed in astrocytomas, as well as other malignant neoplasms [27,28], as seen in our astrocytoma cell line. Given the overexpression of cyclin D1 and nuclear localization of fukutin, we predicted that fukutin may be implicated in cell proliferation.

To clarify implications for the expression status of fukutin in cell proliferation ability, we compared the cell count and Ki-67 labeling index of 1321N1 cells between the fukutin knockdown and overexpression groups. The fact that both the cell count and the Ki-67 labeling index were significantly reduced by fukutin knockdown and significantly increased by fukutin overexpression provided in vitro evidence that fukutin enhances cell proliferation ability. Then, we focused on cyclin D1, a key molecule for cell proliferation. Interestingly, the changing patterns of cyclin D1 mRNA and protein expression levels caused by the expression status of fukutin bore close resemblance to those of the cell count and Ki-67 labeling index. This indicates that fukutin exerts a positive effect on cell proliferation via cyclin D1. There is no precedent except for a previous study suggestive of such characteristics in HeLa cells and breast cancer cells [10].

Physiologically, cyclin D1 is an essential subunit of the cell-cycle engine “cyclin D1-cdk4/6 complex” that drives the transition from phase G1 to phase S, a rate-limiting step through the cell-cycle processes [14,15]. Conventionally, it is known that cyclin D1 expression is upregulated during activation of the mitogen-activated protein kinase (MAPK) pathway, via extracellular signal-regulated kinase (ERK), and that the maximum level of cyclin D1 is responsible for the phase G1 to S shift [29]. Thus, it is evident that cyclin D1 acts as a nuclear sensor of extracellular signals [30]. Overexpression of cyclin D1 is often found in malignant neoplasms [27]. A study demonstrated that cyclin D1 is overexpressed in histologically high-grade gliomas (WHO), and that the higher expression level is positively correlated with poor prognosis [28]. Our observations of the fukutin knockdown-driven decreases in cell proliferation ability and cyclin D1 expression level in 1321N1 cells highlight that fukutin enables astrocytoma cells to proliferate.

A growing body of evidence suggests the involvement of transcription factors in upregulating cyclin D1 expression. Several investigators identified a series of transcription factors for cyclin D1, including AP-1, cAMP response element-binding protein (CREB), and nuclear factor-kappa B (NF-κB) [16]; all of them can bind to the promoter region of cyclin D1. It has been shown that AP-1 binds to the 5′-sided one-third portion of the cyclin D1 promoter region [16]. Considering this background, we narrowed down the fukutin-affinitive target on the promoter by ELISA, using DNA probes designated to the entire portion, the 5′-sided one-third portion, and the AP-1 binding site. Consequently, we found that fukutin can form complexes with the three targets. These observations indicate that fukutin has a potent affinity to AP-1 binding site. At that point, it, however, remained to be determined whether fukutin may bind to AP-1 binding site or AP-1 itself. To address this issue, we searched for the affinity between fukutin and AP-1.

AP-1 is a heterodimeric transcription factor composed of c-Fos and c-Jun [17,18,19,20]. JNK catalyzes the phosphorylation of c-Jun to produce p-c-Jun, leading to AP-1 activation [19]. Then, we employed sandwich ELISA and immunoprecipitant Western blotting in order to determine the relationship between fukutin and c-Jun or p-c-Jun. As a result, these two analyses provided evidence that fukutin can bind not only to c-Jun but also to p-c-Jun. Here, the question of whether fukutin itself is a transcription factor or not surfaced. The former appears to be unlikely, since fukutin does not have amino-acid sequences characteristic of a nuclear localization signal, examined by PSORT II prediction. Another possibility is that fukutin may be a cofactor. In general, it is known that the function of transcription factors is regulated by a balance of cofactors, such as coactivators and corepressors, on transcriptional processes [31]. There are three classes of cofactors in the processes: (i) CREB-binding protein (CREBBP), (ii) c-Jun activation domain-binding protein 1 (JAB1), and (iii) ATP-dependent chromatin remodeling complexes of Switch/sucrose nonfermentable (SWI/SNF) mammalian homologs BRG1 or BRM [32,33]. JNK catalyzes the conversion of c-Jun to p-c-Jun, which enables AP-1 to increase the affinity to CREBBP and the transcription activity [34,35,36]. Concretely, it has been shown that phosphorylation of c-Jun at serine 63/73 residues is required for transcriptional activation of cyclin D1 [37]. This is supported by our finding that JNK inhibitor treatment resulted in the downregulation of cyclin D1 expression. On the other hand, JAB1 activity is not influenced by JNK-mediated phosphorylation of c-Jun. JAB1 interacts with c-Jun to induce complex formation between the AP-1 binding site and c-Jun, enhancing the transcriptional activity [38]. Our study using 1321N1 cells demonstrated that neither knockdown nor overexpression of fukutin affected p-JNK and p-c-Jun level. As our results indicated, it is likely that fukutin is a coactivator for AP-1. However, it has not yet been identified whether there is a common amino-acid sequence between fukutin and the existing coactivators of AP-1. Further investigations are required to conclude that fukutin is a coactivator for AP-1 as a transcription factor of cyclin D1. In addition, our study using 1321N1 cells demonstrated that neither knockdown nor overexpression of fukutin affected p-JNK and p-c-Jun levels. On the other hand, this is inconsistent with a previous study showing that fukutin knockdown in HeLa cells resulted in altered phosphorylation of c-Jun [10]. These observations may reflect a difference in the fukutin-JNK interaction among cell types.

**Figure 8:**
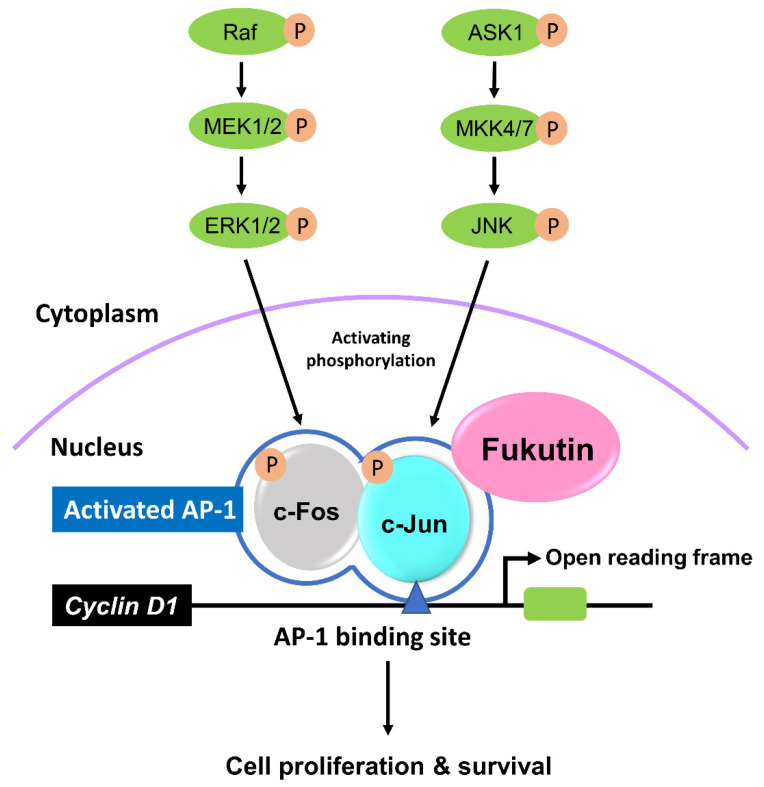
A schematic diagram showing proposed mechanisms of the fukutin-related cyclin D1 induction pathway. AP-1, activator protein-1; ASK1, apoptosis signal-regulating kinase 1; ERK1/2, extracellular signal-regulated protein kinase 1/2; JNK, c-Jun N-terminus kinase; MEK1/2, extracellular signal-regulated protein kinase kinase; MKK4/7, c-Jun N-terminus kinase kinase.

Taken together, we here propose the fukutin/AP-1/cyclin D1 axis, in which fukutin is likely to act as a coactivator for AP-1. This is supported by nuclear localization of fukutin on fluorescence immunocytochemistry. The proposed mechanism is summarized in Figure 8. To the best our knowledge, this is the first report that documents a novel property of fukutin in cultured astrocytes. However, it should be considered with the caveat that fukutin is not always necessary for proliferation of astrocytes, because superficial gliosis occurs in the CNS of patients with FCMD, an autosomal-recessive disorder with homozygous missense mutations in fukutin. Interestingly, CNS lesions in FCMD patients exhibit fibrillary gliosis, rather than hypertrophic cellular astrocytosis, at the brain surface where the glia limitans is abnormal [39], suggesting mild and persistent tissue repair associated with polymicrogyria in this disease. Moreover, there is no precedent describing the incidence of malignant neoplasms such as gliomas in FCMD patients, and it is unclear whether fukutin overexpression is closely relevant to tumorigenesis in the brain. The present observations bring up the involvement of fukutin in cell proliferation ability independent of α-DG glycosylation in astrocytes. However, the entire mechanisms via which fukutin induces cell proliferation remain to be determined. Answers to this and other questions for ultimate significance of multifunctional roles of fukutin require further investigations.

## 4. Materials and Methods

### 4.1. Cell Culture and Experimental Design

The human astrocytoma cell line (1321N1), established by Macintyre [40], was purchased from the European Collection of Cell Cultures (Wiltshire, UK). The 1321N1 cells were grown in Dulbecco’s modified Eagle’s medium (DMEM) (Thermo Fisher Scientific, Waltham, MA, USA) supplemented with 10% fetal bovine serum (Thermo Fisher Scientific) and 1% penicillin–streptomycin (Thermo Fisher Scientific). Cells were maintained at 37 °C in a humidified incubator under 5% CO_2_ atmosphere. The 1321N1 cells were divided into the vehicle group and other groups with or without several treatments mentioned later. The number of cells was counted by the trypan blue dye exclusion method using a TC20™ automated cell counter (Bio-Rad Laboratories, Hercules, CA, USA) on counting slides (Bio-Rad).

### 4.2. Knockdown Fukutin

RNAi was performed according to the previous study [10]. Stealth short hairpin RNA (siRNA) duplexes were designated against fukutin mRNA and synthesized by Thermo Fisher Scientific. The target sense for fukutin mRNA was 5′–UUUGAAGGGAACAAAUUUCCUGUC–3′ (F697). As the scrambled group, Silencer Negative Control No. 1 siRNA (SNC, AM4611) was used, and omission of siRNA was used as vehicle. Fukutin knockdown was confirmed by qPCR (Supplementary Materials Figure S3). 1321N1 cells were plated at a density of approximately 200,000 cells in each 35 mm dish one day prior to the RNA interference (RNAi). siRNA at a final concentration of 40 nM was transfected into the cells using a lipofectamine MAX (Thermo Fisher of Scientific) and Opti-MEM (Thermo Fisher Scientific) according to the manufacturer’s instructions. Cells were harvested 4 days after the RNAi treatment mentioned above.

### 4.3. Overexpression of Fukutin

The open reading frame of fukutin cDNA construct was conjugated to pcDNATM3.1 + DYK (OHu23291) by GenScript (Piscataway, NJ, USA). The plasmids, harboring a large amount of fukutin cDNA, were transfected into 1321N21 cells using lipofectamine 3000 and Opti-MEM, both of which were purchased from Thermo Fisher Scientific. Green-fluorescence protein (GFP)-labeled pcDNA3.1 was used as a negative control. A successful transfection was proven by immunofluorescence for a tag peptide DYKDDDDK and luminescence for GFP.

### 4.4. JNK Inhibition

The JNK inhibitor SP600125 (Abcam, Cambridge, UK) was diluted in dimethyl sulfoxide (DMSO). Semiconfluent 1321N1 cells were incubated for 5, 10, 15, or 30 min with the JNK inhibitor solution at a final concentration of 100 μM, according to the manufacturer’s instructions. Total cell lysate was obtained as mentioned above.

### 4.5. Immunocytochemistry on Cell Block Sections

1321N1 cells were detached using a cell scraper (Iwaki, Tokyo, Japan), fixed in 4% paraformaldehyde/phosphate-buffered saline (PBS) solution (pH 7.6) for 30 min at 4 °C, and then collected by centrifugation. Cells, gathered with 2% agarose, were embedded in paraffin in a routine manner to form blocks. Multiple 3 μm thick sections were deparaffinized and rehydrated. Antigen retrieval was conducted for Ki-67 by microwaving in 10 mM citrate buffer (pH 6) for 20 min, and for cyclin D1 by microwaving in 1 mM ethylenediaminetetraacetic acid (EDTA)/tris (hydroxymethyl) aminomethane (Tris) buffer (pH 9.0) for 40 min. Then, sections were quenched with 3% H_2_O_2_ for 10 min at room temperature (RT) to inhibit endogenous peroxidase activity, rinsed in PBS, pretreated with 5% skim milk/PBS solution for 30 min at RT for blocking nonspecific antibody binding, and subsequently incubated overnight at 4 °C with a mouse monoclonal anti-Ki-67 antibody (clone NCL-Ki67p, Leica Biosystems, Buffalo Groves, IL, USA; 1:2000) and a rabbit monoclonal anti-cyclin D1 antibody (Cat. No. ab134175, Abcam 1:200) Immunoreaction product deposits were visualized by the polymer immunocomplex method using a Histofine Simple Stain Polymer (Nichirei, Tokyo, Japan) for 30 min at RT. The chromogen was 3,3′-diaminobenzidine tetrahydrochloride (Dojindo, Kumamoto, Japan), while hematoxylin was the counterstain. Sections from which the primary antibodies were omitted served as negative reaction controls.

### 4.6. Immunocytochemistry on Culture Slides

1321N1 cells on culture slides (Falcon; Corning, One Riverfront Plaza, NY, USA) were fixed in 95% methanol for 15 min at RT, pretreated with 0.2% Triton X-100 for 15 min, treated with 5% skim milk for 30 min at RT, and subsequently incubated overnight at 4 °C with a mouse monoclonal anti-cyclin D1 antibody (clone DSC-6, Thermo Fisher Scientific; 1:500) followed by Alexa Fluor^®^ 488-conjugated donkey anti-mouse IgG H + L (Thermo Fisher Scientific; 1:500) for 1 h at RT. The nuclei were counterstained with 4′,6-diamidino-2-phenylindole (DAPI). Slides processed with omission of the primary antibody gave a negative reaction control. Immunostained slides were observed using a fluorescence microscope (Nikon ECLIPSE TS100; Nikon, Tokyo, Japan).

### 4.7. Semiquantitative RT-PCR Analysis

Total RNA of 1321N1 cells was extracted using a PureLink RNA Mini Kit (Thermo Fisher Scientific) and reverse-transcribed using an iScript RT Supermix for RT-qPCR (Bio-Rad) to obtain cDNA, which was amplified by an AccuPrime Taq DNA Polymerase System (Thermo Fisher Scientific) using oligonucleotide primer sets. A human cyclin D1-targeted primer set, designed for 159–1046 bp (sense, 5′–ATGGAACACCAGCTCCTGTGCTGCGAA–3′; antisense, 5′–TCAGATGTCCACGTCCCGCACGTCGGT–3′) was synthesized by FASMAC (Atsugi, Japan). A human housekeeping gene for a β-glucuronidase (GUSB)-targeted primer set was purchased from TaKaRa (Tokyo, Japan). The reaction mixture was amplified for 35 cycles in a MJ Research DTC-200 DNA Gradient Peltier Thermal Cycler (MJ JAPAN, Tokyo, Japan). The amplification profile consisted of denaturation for 1.5 min at 94 °C, annealing for 1.5 min at 62 °C, and extension for 1 min at 72 °C. The PCR products were electrophoresed in a 1.2% agarose gel, which was in turn treated with ethidium bromide and photographed under ultraviolet using a ChemiDoc XRS Plus (Bio-Rad). The optical density of each band was taken and measured using an Image Lab (Bio-Rad), and the cyclin D1/GUSB optical density ratio was calculated.

### 4.8. Protein Extraction

To obtain total cell lysate, 1321N1 cells were collected and suspended with lysis buffer, consisting of 50 mM Tris-HCl (pH 7.4), 150 mM NaCl, 1 mM EDTA, 1% Triton X-100, protease inhibitor cocktail (Complete Mini; Roche Diagnostics, Mannheim, Germany), and phosphatase inhibitor cocktail (PhosSTOP; Roche Diagnostics) for 30 min at 4 °C. The samples were centrifuged at 13,500× *g* for 40 min at 4 °C, and the supernatant was used. Cytoplasmic and nuclear protein fractions were isolated extracted separately, using an NE-PER Nuclear and Cytoplasmic Extraction Reagents (Thermo Fisher Scientific) according to the manufacturer’s protocol. The obtained protein extracts were used for Western blot analysis, ELISA, and sandwich ELISA.

### 4.9. Immunoprecipitation

Prior to the immunoprecipitation, a mouse monoclonal anti-c-Jun antibody (Cat. No. sc-74543, 1:20; Santa Cruz Biotechnology, Santa Cruz, CA, USA) was diluted in 0.02% Tween-20/PBS solution (T-PBS), supplemented with SureBeads Protein G-conjugated magnetic beads (Bio-Rad) and incubated for 1 h at RT under a condition with rotating and shaking. After washing the magnetic beads with 0.02% T-PBS, whole-cell lysate protein extracts mentioned above were immunoprecipitated for 1 h at RT with the beads by rotation and shaking. Then, the cell lysate/magnetic beads mixture was washed with 0.02% T-PBS. Then, the mixture was incubated with 4NuPAGE LDS Sample Buffer (4×) (Thermo Fisher Scientific) followed by adding NuPAGE Sample Reducing Agent (10×) (Thermo Fisher Scientific), and denatured for 10 min at 70 °C. The obtained immunoprecipitants were used for Western blot analysis.

### 4.10. Western Blot Analysis

Protein samples (aliquot of 30 μg per lane) were electrophoresed on a 4–20% Mini-PROTEAN TGX Gel (Bio-Rad) and electro-transferred to a polyvinylidene difluoride membrane (Trans-Blot Turbo 0.2 micron PVDF membrane; Bio-Rad). Blotted membranes were treated with a Can Get Signal/PVDF Blocking Reagent (TOYOBO, Tokyo, Japan) for 30 min, washed with Tris-buffered saline (TBS) (10 × TBS #1706435; Bio-Rad) containing 0.02% Tween-20 (T-TBS), and incubated overnight at 4 °C with the primary antibodies diluted in Can Get Signal Solution 1 (TOYOBO). Blots were then rinsed in 0.02% T-TBS and incubated for 1 h at RT with the respective secondary antibodies diluted in Can Get Signal Solution 2 (TOYOBO). Immunoreactive signals were visualized by the chemiluminescence method using an ECL kit (Chemilucent Plus, Merck Millipore, Burlington, MA, USA) and photographed using a ChemiDoc XRS Plus (Bio-Rad). The optical density of each signal band was quantitatively measured using an Image Lab (Bio-Rad). The primary antibodies used were a rabbit polyclonal anti-fukutin antibody (Cat. No. GTX110302, Genetex, Irvine, CA, USA; 1:500), a mouse monoclonal anti-cyclin D1 antibody (clone sc-20044, Santa Cruz Biotechnology;1:500), a rabbit polyclonal anti-c-Jun antibody (Cat. No. sc-1694, Santa Cruz Biotechnology; 1:500), a mouse monoclonal anti-phosphorylated c-Jun (p-c-Jun) antibody (clone sc-822, Santa Cruz; 1:500), a mouse monoclonal anti-c-Jun N-terminus kinase (JNK) antibody (clone sc-7345, Santa Cruz Biotechnology; 1:500), a mouse monoclonal anti-phosphorylated JNK (p-JNK) antibody (clone sc-6254, Santa Cruz Biotechnology; 1:500), a mouse monoclonal anti-lamin A/C antibody (clone 4C11, Cell Signaling Technology, Danvers, MA, USA; 1:5000), and a rabbit polyclonal anti-glyceraldehyde 3-phosphatedehydrogenase (GAPDH) antibody (clone 14C10, Cell Signaling Technology; 1:3000). Blots processed by omitting the primary antibodies were used as negative reaction controls. Both Lamin A and Lamin C were used for quantification.

### 4.11. Preparation of DNA Probes for ELISA-Based Assay

Oligonucleotide primer sets for cyclin D1 promoter entire region (sense, 5′–GTTTAATTGATAATTGTTCT–3′; antisense, 5′–CGGCTCTCGCTTCTGCTGCC–3′) and 5′-sided one-third portion of the cyclin D1 promoter region (sense, 5′–GTTTAATTGATAATTGTTCT–3′; antisense, 5′–AACCGGGAGAAACACACCTC–3′) were synthesized by Thermo Fisher Scientific. Total DNA was extracted by the phenol/chloroform precipitation method. The DNA extracts were subjected to PCR using an AccuPrime Taq DNA Polymerase System (Thermo Fisher Scientific). The targeted region in the reaction mixture was amplified for 35 cycles in an MJ Research DTC-200 DNA Gradient Peltier Thermal Cycler (MJ JAPAN). The amplification profile consisted of denaturation for 1.5 min at 94 °C, annealing for 1.5 min at 62 °C, and extension for 1 min at 72 °C. The PCR products were electrophoresed on a 1.2% agarose gel to identify a 1250 bp DNA fragment for the entire portion and a fragment for the one-third portion. The DNA fragments were subjected to PCR using a PCR DIG Labeling Mix (Roche Diagnostics) and Takara Ex Taq (Takara) to obtain a digoxigenin-labeled double-stranded DNA probes. Similarly, a DNA probe targeted to the AP-1 binding site of cyclin D1 promoter was synthesized with FASMAC, using the sequences (sense, 5′–AATGAGTCAGAATGGAGATCACTGT–3′; antisense, 5′–ACAGTGATCTCCATTCTGACTCATT–3′).

### 4.12. ELISA

Microtiter plates were incubated for 1 h at 37 °C with the anti-fukutin antibody at a dilution of 1:500 followed by washing with TBS. Subsequently, lysis buffer-extracted whole-lysate protein from 1321N21 cells was applied overnight at 42 °C on a microplate at graded concentrations of 0, 0.2, 2, and 20 mg/mL. After washing in TBS, microplates were treated for 1 h at RT with 3% skim milk/PBS solution followed by incubating with alkaline phosphatase-conjugated anti-digoxigenin IgG (Sigma-Aldrich, Burlington, MA, USA) at a dilution of 1:1000 for 1 h at RT. A binding signal was detected using an Alkaline Phosphatase Yellow (qNPP) Liquid Substrate System (Sigma-Aldrich). The titer was determined using a Thermo Scientific Multiskan GO Microplate.

### 4.13. Sandwich ELISA

Whole-cell lysate aliquots were loaded on a microplate preincubated with the anti-fukutin antibody as mentioned in the ELISA-based assay. The microplates were washed with TBS followed by incubating overnight at 42 °C with the anti-c-Jun antibody or the anti-p-c-Jun antibody at a dilution of 1:50. After washing with TBS, the plates were pretreated for 1 h at RT with TBS containing 5% skim milk, followed by HRP-conjugated respective secondary antibodies (Vector Laboratories, Burlingame, CA, USA) at a dilution of 1:10 for 1 h at RT. Detecting the titer was conducted as described for ELISA.

### 4.14. Statistics

Values in each group were expressed as the mean ± SEM. The data were compared among three or more groups by one-way analysis of variance (ANOVA) followed by post hoc Bonferroni correction between two groups. Statistical significance was considered when the *p*-value was less than 0.05.

## Data Availability

Not applicable.

## Supplementary Materials

The following are available online at https://www.mdpi.com/article/10.3390/ijms222212153/s1.
