# Supplementary figures and images for: Fukutin Protein Participates in Cell Proliferation by Enhancing Cyclin D1 Expression through Binding to the Transcription Factor Activator Protein-1: An In Vitro Study
Source: Int J Mol Sci. 2021 Nov 10;22(22):12153. doi: 10.3390/ijms222212153 (PMC8622492; doi:10.3390/ijms222212153)

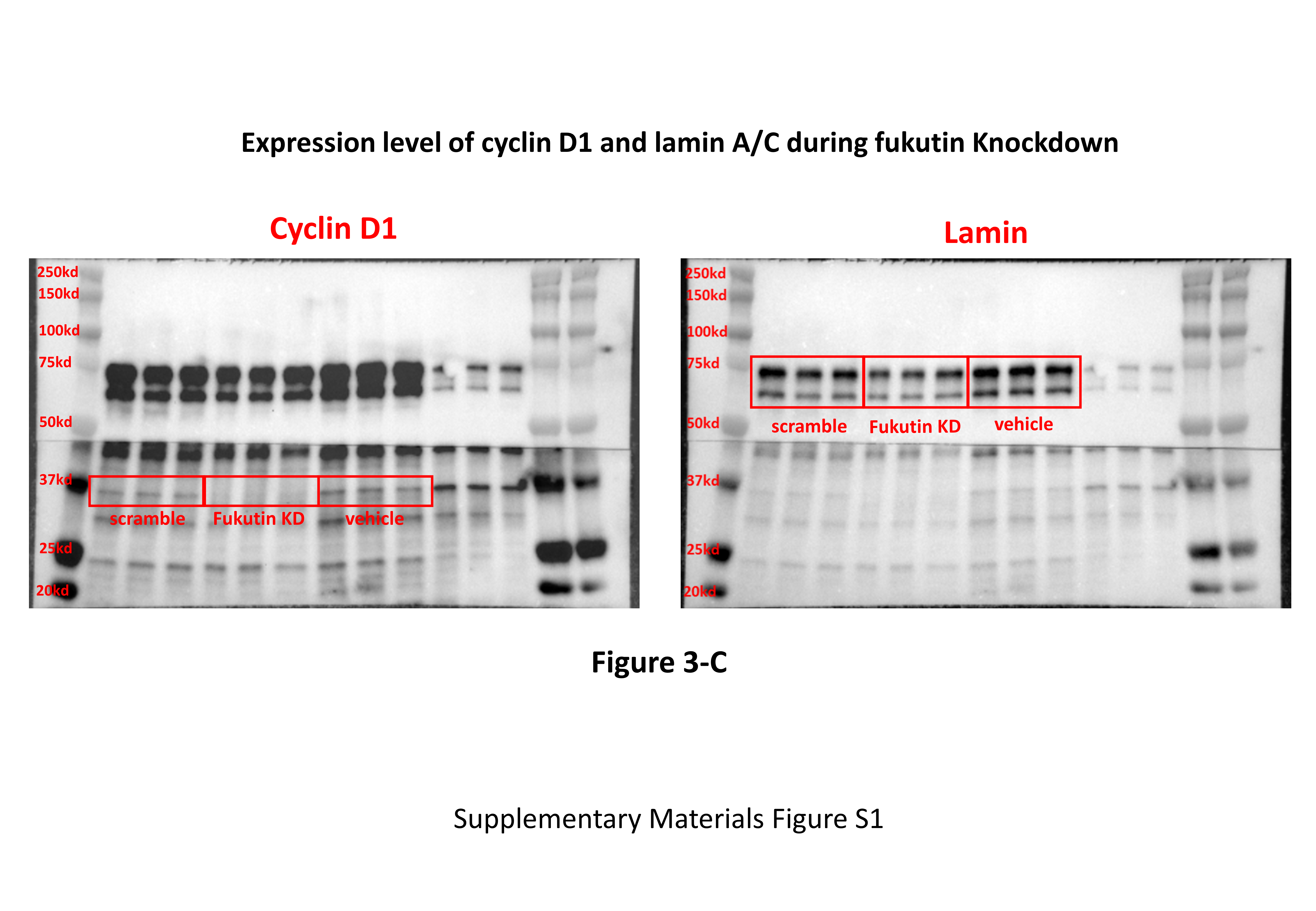

Supplement: Supplementary file 1 [file ijms-22-12153-s001.zip › Figure S1.TIF]

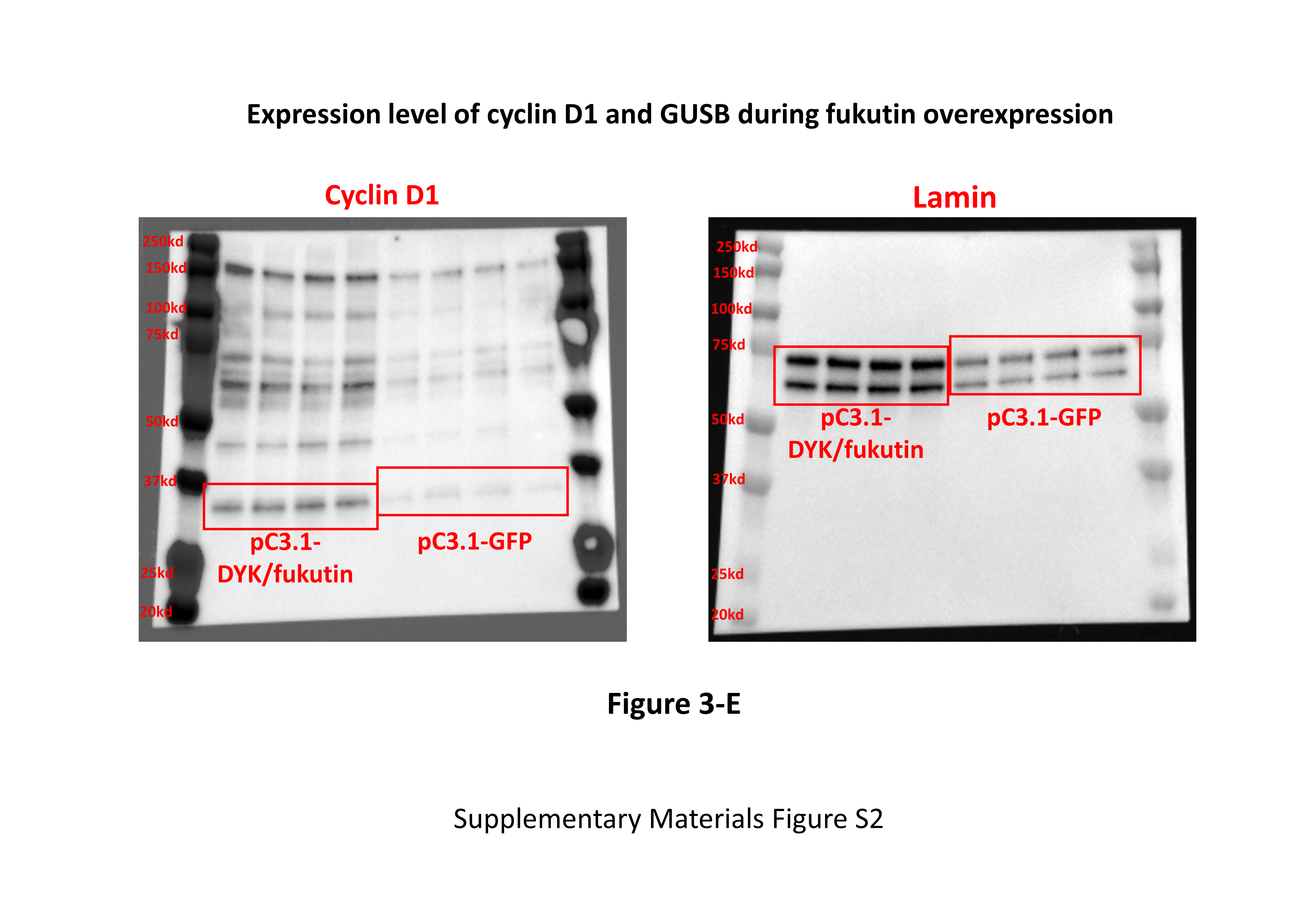

Supplement: Supplementary file 1 [file ijms-22-12153-s001.zip › Figure S2.TIF]

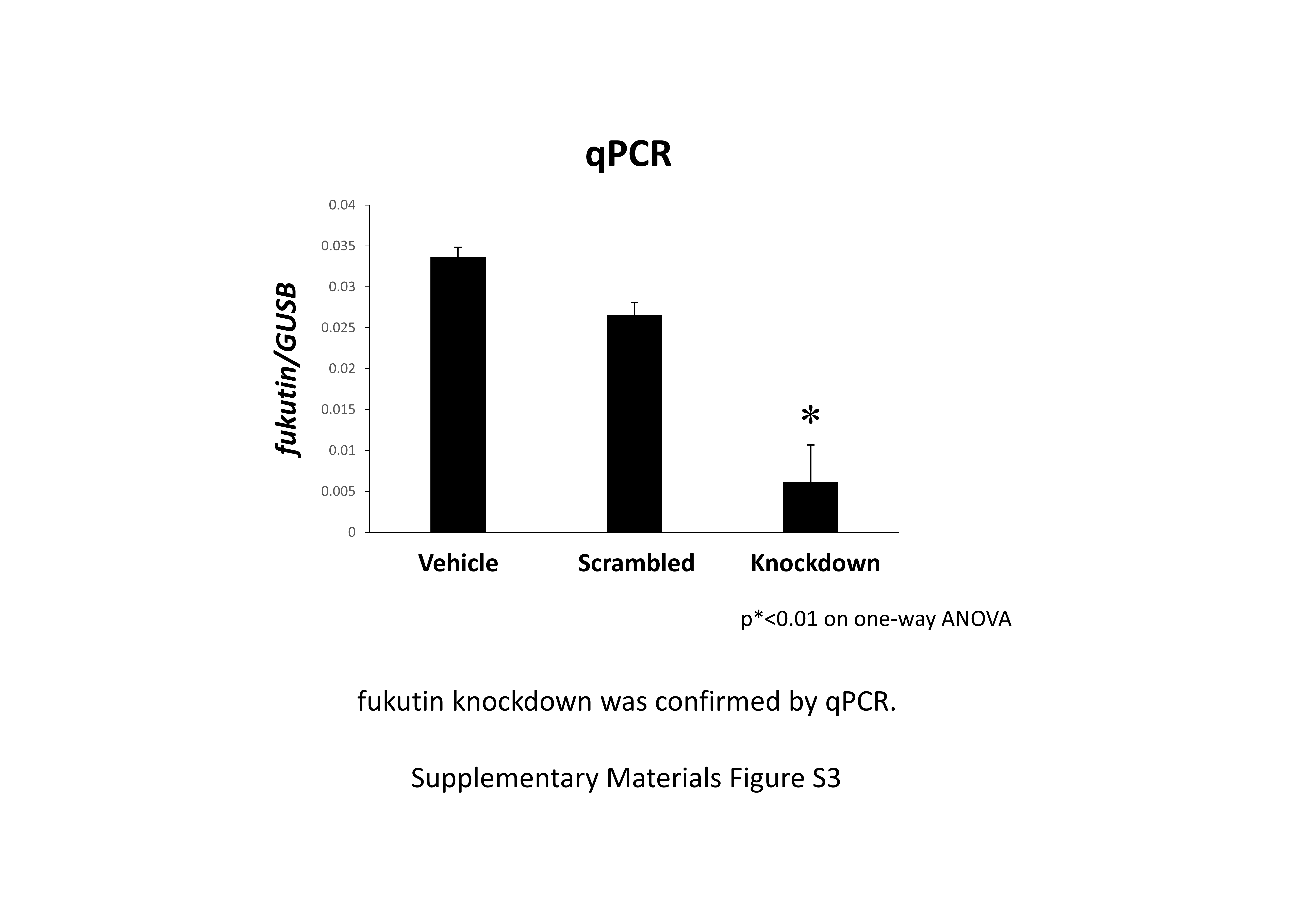

Supplement: Supplementary file 1 [file ijms-22-12153-s001.zip › Figure S3.TIF]
